# Dynamic Balance of Microglia and Astrocytes Involved in the Remyelinating Effect of Ginkgolide B

**DOI:** 10.3389/fncel.2019.00572

**Published:** 2020-01-08

**Authors:** Jun-Jun Yin, Yan He, Jun An, Qiang Miao, Ruo-Xuan Sui, Qing Wang, Jie-Zhong Yu, Bao-Guo Xiao, Cun-Gen Ma

**Affiliations:** ^1^The Key Research Laboratory of Benefiting Qi for Acting Blood Circulation Method to Treat Multiple Sclerosis of State Administration of Traditional Chinese Medicine, Research Center of Neurobiology, Shanxi University of Chinese Medicine, Taiyuan, China; ^2^Shanxi Key Laboratory of Inflammatory Neurodegenerative Diseases, Institute of Brain Science, Shanxi Datong University, Datong, China; ^3^Institute of Neurology, Huashan Hospital, Institutes of Brain Science and State Key Laboratory of Medical Neurobiology, Fudan University, Shanghai, China

**Keywords:** Ginkgolide B, cuprizone-induced demyelination, microglia, astrocytes, remyelination

## Abstract

Multiple sclerosis (MS) is an inflammatory demyelinating disorder in the central nervous system (CNS), in which remyelination failure results in persistent neurologic impairment. Ginkgolide B (GB), a major terpene lactone and active component of Ginkgo biloba, has neuroprotective effects in several models of neurological diseases. Here, our results show, by using an *in vivo* cuprizone (CPZ)-induced demyelinating model, administration of GB improved behavior abnormalities, promoted myelin generation, and significantly regulated the dynamic balance of microglia and astrocytes by inhibiting the expression of TLR4, NF-κB and iNOS as well as IL-1β and TNF-α, and up-regulating the expression of Arg-1 and neurotrophic factors. GB treatment also induced the generation of oligodendrocyte precursor cells (OPCs). *In vitro* cell experiments yielded the results similar to those of the *in vivo* model. The dynamic balance by decreasing microglia-mediated neuroinflammation and promoting astrocyte-derived neurotrophic factors should contribute to endogenous remyelination. Despite GB treatment may represent a novel strategy for promoting myelin recovery, the precise mechanism of GB targeting microglia and astrocytes remains to be further explored.

## Introduction

Multiple sclerosis (MS) is an inflammatory demyelinating disease of the central nervous system (CNS), accompanied by the following pathological manifestations, such as immune cell invasion, demyelination and axonal degeneration (Reich et al., 2018). Although the exact cause of oligodendrocyte damage and loss is not fully elucidated, several hypotheses have been proposed and are being tested, including oxidative stress, mitochondrial dysfunction, neuroinflammation, protein misfolding (Dendrou et al., 2015; Stone and Lin, 2015; Ibitoye et al., 2016; Rajda et al., 2017).

In recent years, there has been a paradigm shift in the treatment of MS because of the approval of some new disease-modifying drugs distinct mechanisms of action (Vaughn et al., 2018). However, the emergence of highly effective treatment options for MS has been accompanied by an increasingly complex array of adverse effects, especially with regard to immune compromise following long-term immune therapy (Klotz et al., 2019). It was reported that Fingolimod is also associated with an increased incidence of other skin malignancies (Velter et al., 2019). Graves’ disease is one of the most frequent presentations when Alemtuzumab is effective in the treatment of relapsing-remitting MS (Alamo et al., 2019). The increasing complexity of MS therapies, especially with regard to treatment sequencing and potential additive immune compromise, heightens the need for more effective and safer therapeutic drugs. In addition, axons and neurons are mostly preserved in early MS, leading to gradual neuroaxonal loss correlating with patient disability (Granberg et al., 2017). The results of recent developments in imaging technology have demonstrated that the neurodegenerative process then gradually becomes self-perpetuating, resulting in irreversible disability (Friese et al., 2014). New therapeutic strategies targeting neuroprotection and remyelination are two unmet needs in the treatment of MS (Thompson et al., 2018).

Ginkgo biloba, a slow-growing tree indigenous to Eastern Asia, is one of the oldest species of trees on the planet, with neuroprotective effects (Ahlemeyer and Krieglstein, 2003). *In vitro*, the extract of Ginkgo biloba protected cultured neurons against death induced by hypoxia, hydrogen peroxide, glutamate, amyloid, nitric oxide, and 1-methyl-4-phenyl-1,2,3,6-tetrahydropyridine (MPTP; Ahlemeyer and Krieglstein, 2003). *In vivo*, the extract of Ginkgo biloba also protected neuron damage and/or death through different mechanisms in middle cerebral artery occlusion (MCAO), focal cerebral ischemia, hypoxia, and amyotrophic lateral sclerosis (ALS) mice or rat models (Ahlemeyer and Krieglstein, 2003). Ginkgolide B (GB), a major terpene lactone and active component of Ginkgo biloba, has neuroprotective effects in several models of neurological diseases. GB alleviated hypoxia-induced neuronal damage in the rat hippocampus by inhibiting oxidative stress and apoptosis (Li et al., 2019) and NLRP3 inflammasome activation (Chen et al., 2018). GB promoted the differentiation of neural stem cells following cerebral ischemia/reperfusion injury (Zheng et al., 2018). Ginkgo biloba extract EGb 761 elicited protective protein clearance through the autophagy-lysosomal pathway in tau-transgenic mice and cultured neurons (Qin et al., 2018). However, there is no experimental information on the therapeutic value of GB for the protection and regeneration of myelin sheaths in the CNS.

CPZ-induced demyelination, unlike experimental autoimmune encephalomyelitis (EAE), is independent of autoimmune attacks and is also often used to mimic the pathology of human MS (Zhen et al., 2017; Vega-Riquer et al., 2019). Remarkably, some events and aspects of the histological pattern induced by CPZ are similar to those found in MS. It is disappointing that a large number of MS patients suffer from the influence of demyelination such as disable and anxiety in spite of the myelin repairing following with demyelination frequently. Therefore, CPZ-induced demyelination provides a good experimental approach to study demyelination and remyelination and is a suitable pharmacological model for developing some promising drugs of neuroprotection and/or remyelination. In this study, we observed the therapeutic potential of GB for myelin protection and regeneration in the CPZ-induced demyelination model and explored the possible cellular and molecular mechanisms of action.

## Materials and Methods

### Animals

Ten-week-old C57BL/6 male mice, weighing 15–20 g, were purchased from Vital River Laboratory Animal Technology Company Limited (Beijing, China) and used for all experiments. Upon arrival, mice were acclimatized and fed for 7 days before starting the experiment. Mice were kept under controlled temperature (25°C) and humidity with normal light/dark cycle conditions throughout the whole experiment. All animal procedures were approved by the Council for Laboratory and Ethics Committee of Shanxi University of Chinese Medicine, Taiyuan, China. All animal protocol was performed according to the International Council for Laboratory Animal Science guidelines.

### CPZ-Induced Demyelinating Model and Administration of GB

The experimental mice were randomly divided into three groups: normal diet group (Normal, *n* = 8), CPZ diet group (CPZ, *n* = 8) and CPZ diet plus GB intervention group (CPZ + GB, *n* = 7). To induce demyelination, mice included in normal and CPZ groups were fed with 0.2% (w/w) cuprizone (CPZ; Sigma-Aldrich, USA) in chow diet *ad libitum* for a total of 6 weeks. After 4 weeks, experimental mice were intraperitoneally (i.p.) injected with GB (20 mg/kg) or normal saline (NS) for consecutive 14 days. One mouse in the CPZ + GB group died of unknown causes on the 7th day after feeding CPZ. No other adverse events occurred in this study.

### Behavior Test

It has been reported that demyelinating lesions are indicative of anxiety-and depression-like behavior and cognitive impairment. Therefore, forced swimming (FS), elevated plus maze (EPM), and T-maze (TM) tests were performed for anxiety, depression and cognitive impairment on the day before the end of the experiment. All behavioral tests were repeated three times in a separate cohort of mice. For EPM, the mice were placed individually in the center of the plus-maze facing an open arm. The number of entering closed arms was recorded during the 10-min testing period. Distance in the open arm and the number of entries into the open arm were recorded. For FST, the mice were placed individually to swim in a plastic cylinder (height: 30 cm, diameter: 10 cm) filled with 20 cm of 25 ± 1°C water. Cumulative activity distance and total resting time were recorded during 1 min. The TM consisted of two arms and one stem. There was a start box on the bottom of the stem of the maze. Two target compartments were located at the end of both arms of the maze. Mice were tested 10 times per day for 3 days. Mice were positioned at the end of one stem and given the possibility to move for 10 min. Resting time in food arm zone and number of entry into food arm were recorded. All data acquisition and analysis were performed automatically using digital video and Image™ software.

### Tissue Preparation

After saline infusion and fixation with 10% chloralhydrate, the brain (*n* = 3–4) was carefully removed, immersed in 30% sucrose solution for 24 h, and then stored at −80°C for subsequent immunohistochemistry. The other half of the mice (*n* = 4) only received a saline infusion, and the brain was removed and stored at −80°C for subsequent enzyme-linked immunosorbent assay (ELISA) and western blot assays.

### Myelin Staining

Histological myelin staining was performed by Luxol Fast Blue (LFB) staining and Black Gold II staining. LFB staining: the slides were stained in LFB at 56°C overnight. After washing with 95% ethanol and distilled water, the color was differentiated in lithium carbonate solution for 15 s followed by distilled water and three washes of 80% alcohol. Black Gold II staining: the slides were dehydrated for 60 min on a slide warmer and then rehydrated with purified water. Pre-warmed Black Gold II solution was added onto sections and incubated at 60°C for 15 min. After washing with Milli-Q water, pre-warmed 1% sodium thiosulfate was added to the slides and incubated for 3 min, followed by the incubation with cresyl violet stain for 3 min. The mean optical densities of LFB and Black Gold II staining in the corpus callosum were measured using Image-Pro Plus 6.0 software. Myelin basic protein (MBP) staining: after blocking with 1% BSA/PBS at room temperature (RT) for 30 min, the slides were incubated with anti-MBP (1:500, Abcam, Burlingame, CA, USA) at 4°C for 18 h, and then incubated with anti-rabbit IgG (1:1,000, Abcam, Burlingame, CA, USA) at RT for 2 h. As a negative control, additional sections were treated similarly, but the primary antibodies were omitted. Results were visualized and analyzed under fluorescent microscopy by Image-Pro Plus 6.0 software described by immunohistochemistry in a blinded fashion. Quantification was performed on three sections per mouse.

### Immunohistochemistry

Brain slides were blocked with 1% BSA/PBS for 1 h at RT and incubated with anti-MBP (1:500, Abcam, Burlingame, CA, USA), anti-Iba-1 (1:300, BD Bioscience, USA), anti-NF-κB/p65 (1:200, Cell Signaling Technology, Danvers, MA, USA), anti-TLR4 (1:200, Bioworld Tech. Inc., St. Louis Park, MN, USA), anti-iNOS (1:300, Abcam, Burlingame, CA, USA), anti-Arg-1 (1:300, Gene Tex, Irvine, CA, USA), anti-GFAP (1:250, Thermo Fisher, USA), anti-BDNF (1:200, Novusbio, Centennial, CO, USA), anti-GDNF (1:500, Abcam, Burlingame, CA, USA), anti-NG2 (1:300, Millipore, Germany) and anti-Ki67 (1:200, BD Pharmingen, USA) at 4°C for overnight, followed by corresponding secondary antibodies at RT for 1 h. The results were repeated three times with consecutive brain slices from each group, and the slides were observed under fluorescence microscopy in a blinded fashion. Analysis and quantification were done in three sections/per mouse by Image-Pro Plus 6.0 software.

### Western Blot Analysis

Extracts of protein (30 μg) were separated by sodium dodecyl sulfate-polyacrylamide gel electrophoresis (SDS-PAGE) and subsequently transferred electrophoretically to nitrocellulose membrane (Millipore). For SDS-PAGF, 10% separating gel and 5% stacking gel were used to perform electrophoretic separation. After blocking with 5% skimmed milk at RT for 1 h, the membranes were incubated with rabbit anti-Iba-1 (1:1,000, Abcam, Burlingame, CA, USA), rabbit anti-TLR4(1:500, Abcam, Burlingame, CA, USA), rabbit anti-NFκB (1:800, Abcam, Burlingame, CA, USA), rabbit anti-iNOS (1:800, Gene Tex, Irvine, CA, USA), rabbit anti-Arginase 1 (1:500, Gene Tex, Irvine, CA, USA), rabbit anti-GFAP (1:1,000, Thermo Fisher, USA), rabbit anti-tubulin (1:5,000, Bioword, USA) and rabbit anti-β-actin (1:1,000, Cell Signaling Technology, USA) at 4°C for overnight, followed by HRP-conjugated goat anti-rabbit secondary antibodies (1:1,000, Abcam, Burlingame, CA, USA) at RT for 2 h. Immunoblots were developed with an enhanced chemiluminescence system (GE Healthcare Life Sciences) and measured using Quantity Software (Bio-Rad, Hercules, CA, USA). Tublin and β-actin were used as an internal reference. The experiment was repeated at least three times.

### Enzyme-Linked Immunosorbent Assay (ELISA)

The concentrations of IFN-γ, TNF-α, BDNF, GDNF in brain homogenates were measured by sandwich ELISA kit (R&D system, USA) according to the manufacturer’s instructions. ELISA procedure should be done at RT but the standard dilutions kept on ice while during preparation. We use pg/ml to express the results.

### Cell Culture

#### Astrocytes

Primary astrocytes were prepared from postnatal (1-day-old) C57BL/6 mice. Under aseptic conditions, cerebral cortices were dissected and placed in sterile phenol red-free Dulbecco’s Modified Eagle’s Medium (DMEM, 4,500 mg/l Glucose, 4 mM L-Glutamine, 1 mM Sodium Pyruvate, Thermo Scientific) containing 1% of streptomycin (10,000 μg/ml)-penicillin (10,000 units/ml). The cells were dissociated into a single cell suspension with a Pasteur pipette, and then seeded in 6-well culture plates at a density of 2.5 × 10^5^ cells/well in a humidified incubator at 37°C and 5% CO_2_. When mixed glial cultures reached 90% confluence (typically 8–10 days), the microglia in the cultures were removed by shaking constantly for 4–6 h. Immunocytochemical staining and quantitative PCR were measured by using cultured primary astrocytes.

#### Microglia

The BV2 mouse microglia cell line (from ShenKe Biological Technology Company Limited, Shanghai, China) was cultured in Dulbecco’s modified Eagle medium (DMEM, Gibco, Gaithersburg, MD, USA) with 10% fetal bovine serum, 100 μ/ml penicillin, and 100 mg/ml streptomycin (complete medium) at 37°C and a humidified atmosphere augmented with 5% CO_2_. Cells were plated at a density of 1 × 10^5^ /ml for all experiments. The BV2 was stimulated with LPS at a concentration of 1 μg/ml and the stimulation time was 12 h.

#### Oligodendrocytes

Oligodendrocytes were isolated from the brain of newborn mice (1-day-old) by CD140a MicroBead kit (Miltenyi Biotec) according to product instructions. Cells were plated at a density of 1 × 10^5^ /ml for all experiments. PDGFRα was expressed in sorted cells.

### Immunocytochemistry

Cells were blocked with 1% BSA/PBS for 1 h at RT and incubated with anti-Iba-1 (1:300, BD Bioscience, San Jose, CA, USA), anti-NF-κB/p65 (1:200, Cell Signaling Technology, USA), anti-iNOS (1:300, Abcam, Burlingame, CA, USA), anti-Arg-1 (1:300, Gene Tex, Irvine, CA, USA), anti-GFAP (1:250, Thermo Fisher, USA), anti-BDNF (1:200, Novusbio, Centennial, CO, USA), anti-GDNF (1:500, Abcam, USA), anti-PDGFRα (1:300, Millipore, Germany) and anti-Ki67 (1:200, BD Pharmingen, USA) at 4°C for overnight, followed by corresponding secondary antibodies at RT for 1 h. The results were repeated three times with similar results. Analysis and quantification were done by Image-Pro Plus 6.0 software.

### Quantitative RT-PCR

Cells were lysed in Buffer RL, and the total RNA was isolated by RNAprep Pure cell Kit. The total RNA yield and concentration were measured by using Thermo(N_ANO_D_ROP_ O_NE_). cDNA was then generated using the Prime Script™ RT Master Mix (Takara Bio, Japan), according to the manufacturer’s instruction. Quantitative real-time PCR was performed by using TB Green™ Premix Ex Taq™ II (Takara, Japan) on the CFX96™ real-time PCR instrument.

The primers used in this study are as follows: BDNF:(FWD-AGCTCCTGACCTTGGTCTTG, REV-AGACCTCTCGAACCTGCCC), GDNF:(FWD-AAGTGGCACAGTTTTGCTGGA, REV-GCTAACAGTGACATCACACAAGT), iNOS:(FWD-CAGGGAGAACAGTACATGAACAC, REV-TTGGATACACTGCTACAGGGA), Arg-1:(FWD-CTGAGAGATTCAAGGCAAGAGG, REV-GAACGCGCTATCTTACCCCAG), ACTB:(FWD-GTGCTATGTTGCTCTAGACTTCG, REV-ATGCCACAGGATTCCATACC).

Cycle conditions were 95°C for 30 s, followed by 40 cycles of 95°C for 5 s and 60°C for 30 s, and the melt curve was 95°C for 15 s, −60°C for 1 min and −95°C for 15 s.

### Statistical Analysis

Statistical analysis of differences between groups was performed by one-way analysis of variance (ANOVA). All statistical comparisons were performed using GraphPad Prism version 6.0 (GraphPad Software, San Diego, CA, USA). All data are expressed as mean ± SEM. The difference of *p* < 0.05 was considered to be statistically significant.

## Results

### Ginkgolide B Improves Behavior and Promotes Myelin Repair

**Figure 1:**
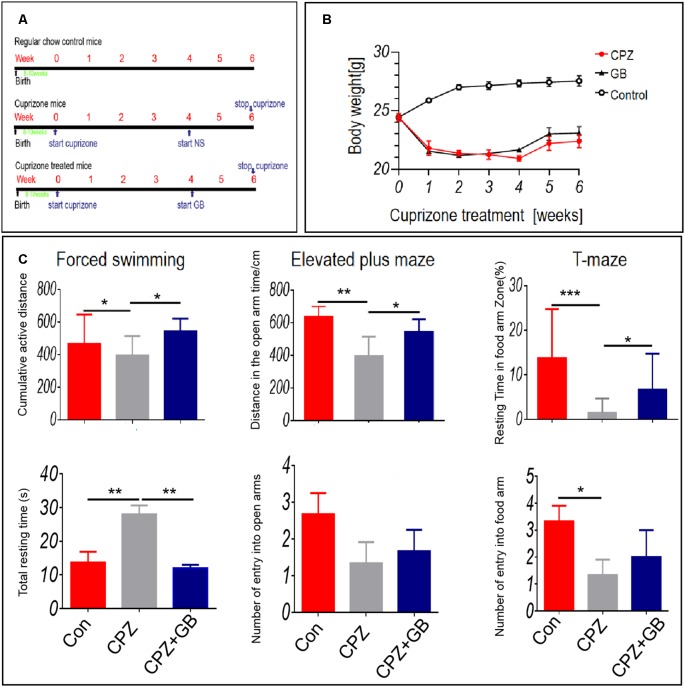
Ginkgolide B (GB) improved behavioral abnormality in cuprizone (CPZ)-induced demyelination. Mice were fed with chow containing 0.2% CPZ for 6 weeks and were intraperitoneally injected with GB for 14 consecutive days from the 4th to 6th week of CPZ feeding. **(A)** The design scheme of experimental protocol (*n* = 8/group), **(B)** body weight change, **(C)** anxiety-and depression-like behavior and cognitive function by forced swimming test (FST), elevated plus maze (EPM) and T-maze (TM) tests. Behavioral results were obtained by video camera and quantified by Image-Pro Plus 6.0 software. The results represent the mean ± SEM. *p < 0.05, **p < 0.01, ***p < 0.001.

The acute demyelinating model was induced by feeding 6 weeks of C57BL/6 mice with a 0.2% CPZ. The experimental design is shown in Figure 1A. One week after CPZ feeding, the weight of mice fed with CPZ was significantly decreased than that of mice fed with normal diet (Figure 1B). Although the weight of mice treated with GB was slightly increased than that of CPZ mice, there was no statistical significance (Figure 1B).

Anxiety- and depression-like behavior and cognitive impairment have been observed in demyelinating lesions (Acharjee et al., 2013; Xu et al., 2019; Zimmermann et al., 2019). In this study, FS, EPM and TM tests were used to evaluate anxiety-and depression-like behavior and cognitive impairment. The results showed that CPZ resulted in above behavioral abnormalities after 6 weeks of CPZ feeding as compared with mice fed with normal diet (Figure 1C, *p* < 0.05, *p* < 0.01 and *p* < 0.001, respectively). However, GB treatment improved effectively these behavioral abnormalities after 2 weeks of treatment (Figure 1C, *p* < 0.05, *p* < 0.01, respectively).

**Figure 2:**
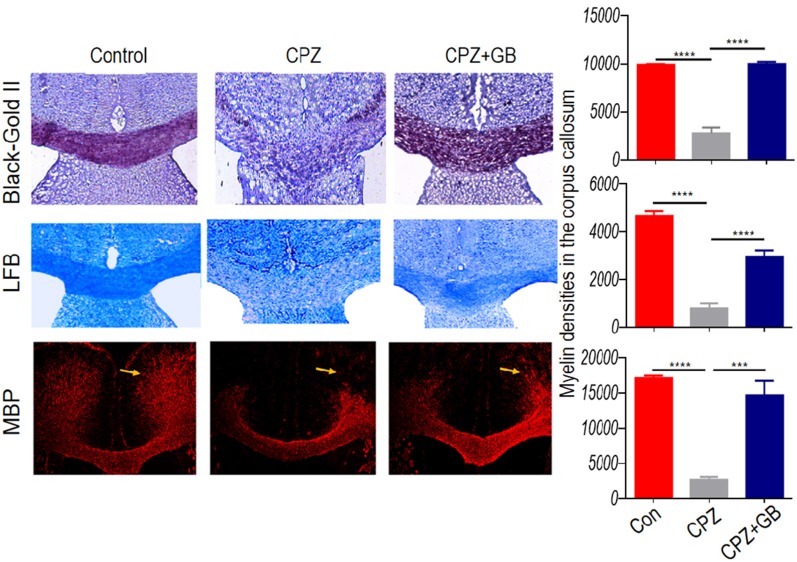
GB promoted remyelination in CPZ-induced demyelination. Mice were fed with chow containing 0.2% CPZ for 6 weeks and were intraperitoneally injected with GB for 14 consecutive days from the 4th to 6th week of CPZ feeding. Histological evaluation of demyelination was carried out by Black Gold II, luxol fast blue (LFB) and myelin basic protein (MBP) staining (*n* = 3–4/each group), and quantified by Image-Pro Plus 6.0 software. Representative images of Black Gold II, LFB and MBP staining are observed in the corpus callosum of the brain. The results represent the mean ± SEM. ***p < 0.001, ****p < 0.0001.

To evaluate the protective effect of GB during demyelination, three methods including Black Gold II, LFB, and MBP staining, were simultaneously used in this study. The results showed that compared with normal mice, the myelin sheath in the corpus callosum of mice fed with CPZ was obviously lost by both Black Gold II and LFB staining (Figure 2, *p* < 0.0001, respectively). GB treatment effectively increased the intensity of Black Gold II and LFB staining (Figure 2, *p* < 0.0001, respectively), suggesting the protective or regenerative role of GB during demyelination. Similarly, MBP staining showed the damage to myelin sheath caused by CPZ feeding (Figure 2, *p* < 0.0001), which could be repaired by GB treatment, especially in the cingulum region (Figure 2, *p* < 0.001).

### Ginkgolide B Reduces Inflammation Associated With Microglia

**Figure 3:**
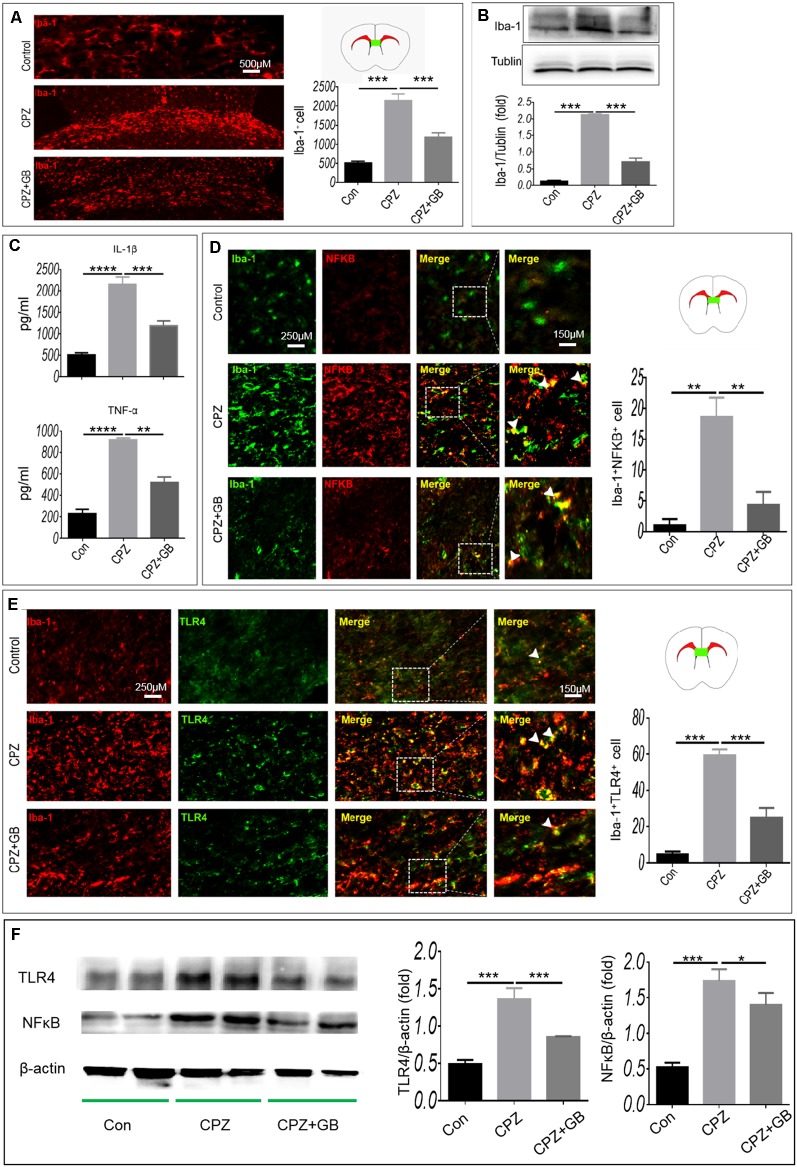
GB inhibited the microglial inflammatory response. Mice were fed with chow containing 0.2% CPZ for 6 weeks and were intraperitoneally injected with GB for 14 consecutive days from the 4th to 6th week of CPZ feeding. **(A)** Iba-1^+^ microglia in the corpus callosum of brain by immunohistochemistry, **(B)** expression of Iba-1^+^ protein in the extract of brain by western blot, **(C)** levels of inflammatory cytokines IL-1β and TNF-α in the extract of brain by enzyme-linked immunosorbent assay (ELISA), **(D)** double immunohistochemical staining with anti-Iba-1 and anti-p-NF-κB/p65 in the corpus callosum, **(E)** double immunohistochemical staining with anti-Iba-1 and anti-TLR4 in the corpus callosum. **(F)** Expression of TLR4 and p-NF-κB/p65 in the extract of the brain by western blot. Representative images are obtained from three to four mice/each group, with similar results. The results represent the mean ± SEM. *p < 0.05, **p < 0.01, ***p < 0.001, ****p < 0.0001.

The CPZ model is characterized by primary and reversible demyelination, accompanied by microglia-mediated neuroinflammation that can aggravate demyelinating lesions in the CPZ model. Compared with normal mice, CPZ-fed mice showed the accumulation of Iba-1^+^ microglia in the corpus callosum (Figure 3A, *p* < 0.001) and the increase of Iba-1 expression in the brain (Figure 3B, *p* < 0.001), suggesting that myelin damage may trigger the migration and enrichment of microglia in the corpus callosum. However, GB treatment obviously declined the accumulation of Iba-1^+^ microglia in the corpus callosum and the increase of Iba-1 expression in the brain (Figures 3A,B, *p* < 0.001, respectively).

It is generally believed that CPZ feeding resulted in the activation of microglia. As shown in Figure 3C, the levels of inflammatory cytokines IL-1β and TNF-α wee elevated in CPZ-fed mice (*p* < 0.0001, respectively), which can be declined by GB treatment (*p* < 0.001, *p* < 0.0001, respectively). Next, we observed the phenotypic characteristics of microglia in the corpus callosum. As shown in Figure 3D, the expression of NF-κB and TLR4 on Iba-1^+^ microglia in CPZ-fed mice was higher than that in normal-fed mice (Figures 3D,E, *p* < 0.01 and *p* < 0.001, respectively). GB treatment inhibited the expression of NF-κB and TLR4 on Iba-1^+^ microglia in CPZ-fed mice (Figures 3D,E, *p* < 0.01 and *p* < 0.001, respectively). The results from Western blot showed that the expression of TLR4 and p-NF-κB/p65 was up-regulated 6 weeks after CPZ feeding (Figure 3F, *p* < 0.001 respectively), and was effectively inhibited by GB intervention (Figure 3F, *p* < 0.001 and *p* < 0.05, respectively), which is consistent with the that of immunohistochemical staining in Figures 3D,E.

**Figure 4:**
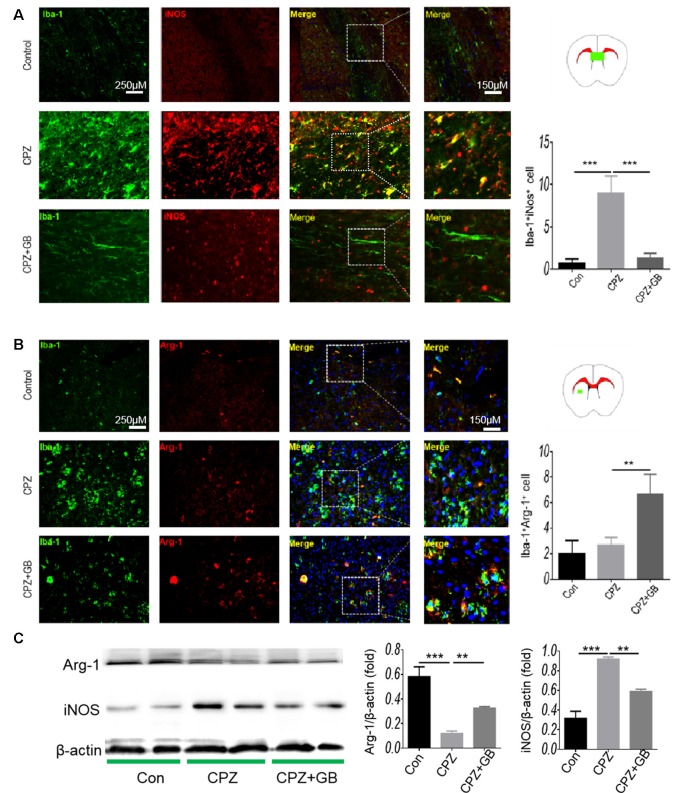
GB influenced the phenotype of M1/M2 microglia. Mice were fed with chow containing 0.2% CPZ for 6 weeks and were intraperitoneally injected with GB for 14 consecutive days from the 4th to 6th week of CPZ feeding. **(A)** Double immunohistochemical staining with anti-Iba-1 and anti-iNOS in the corpus callosum, **(B)** double immunohistochemical staining with anti-Iba-1 and anti-Arg-1 in the striatum. **(C)** Expression of Arg-1 and iNOS protein in the extract of the brain by western blot. Representative images are obtained from three to four mice/each group, with similar results. The results represent the mean ± SEM. **p < 0.01, ***p < 0.001.

iNOS and Arg-1 are two typical markers of M1 and M2 microglia, respectively. We further used immunohistochemistry to detect the expression of iNOS and Arg-1 on Iba-1^+^ microglia. The results showed that Iba-1^+^ microglia expressing iNOS in mice fed with CPZ was significantly higher than that in normal mice (Figure 4A, *p* < 0.001), which was effectively inhibited by GB treatment (Figure 4A, *p* < 0.001). Conversely, CPZ feeding did not decline the expression of Arg-1 on Iba-1^+^ microglia, but which was indeed up-regulated by GB treatment, as compared with CPZ-fed mice (Figure 4B, *p* < 0.01). The results from Western blot showed that the expression of Arg-1 was inhibited and the expression of iNOS was elevated 6 weeks after CPZ feeding (Figure 4C, *p* < 0.001 respectively), and was effectively induced and declined by GB intervention (Figure 4C, *p* < 0.01 respectively), which is consistent with the that of immunohistochemical staining in Figures 4A,B.

**Figure 5:**
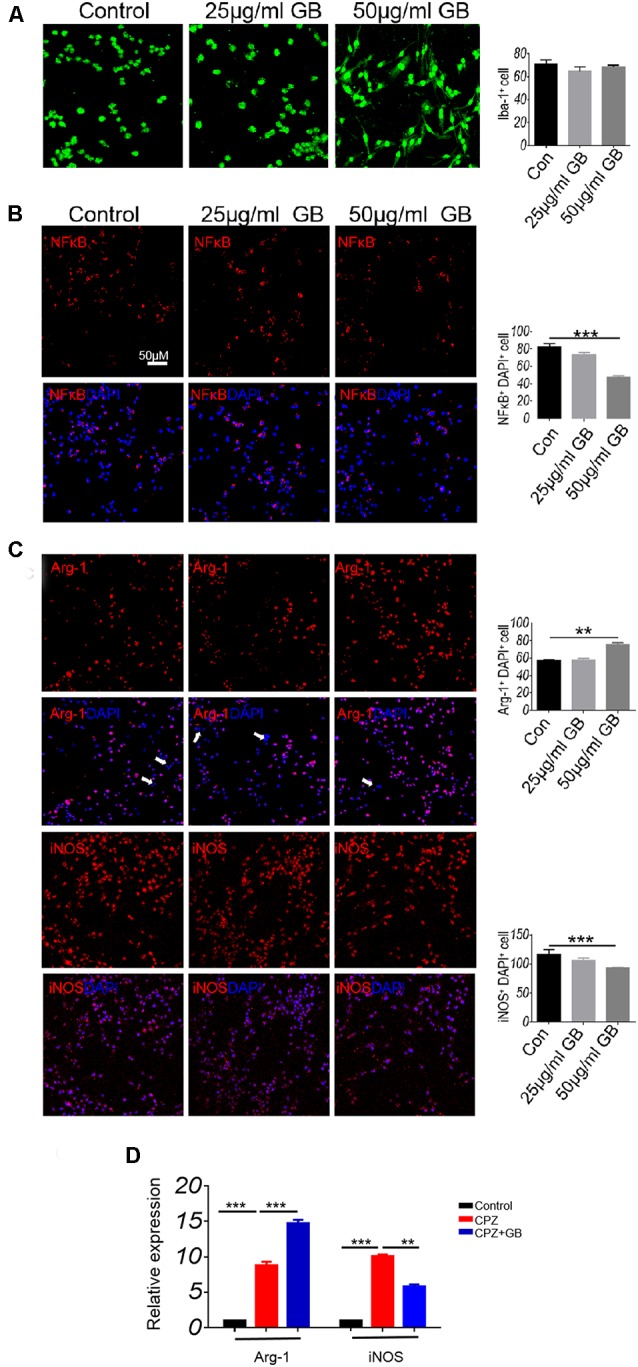
GB influenced the phenotype of M1/M2 in BV2 cells. **(A)** Immunocytochemistry staining with anti-Iba-1 in BV2 microglia. **(B)** Double immunocytochemistry staining with anti-DAPI and anti-p-NF-κB/p65 in the BV2 cells, **(C)** double immunocytochemistry staining with anti-DAPI and anti-Arg-1/anti-iNOS in the BV2 cells. **(D)** Relative expression of Arg-1 and iNOS mRNA in BV2 microglia by qPCR. Representative images are obtained from three replicate samples/each group, with similar results. The results represent the mean ± SEM. **p < 0.01, ***p < 0.001.

*In vitro* cell experiments, Iba-1 was expressed in cultured BV2 microglia (Figure 5A). The results showed that high concentration of GB (50 μg/ml) inhibited LPS-induced the expression of p-NF-κB/p65 (Figure 5B, *p* < 0.001), decreased iNOS expression (Figure 5C, *p* < 0.01) and increased Arg-1 expression (Figure 5C, *p* < 0.001) compared with control. The results from qPCR showed that relative expression of Arg-1 and iNOS mRNA was up-regulated 6 weeks after CPZ feeding (Figure 5D, *p* < 0.01 and *p* < 0.001, respectively). GB intervention further increased the relative expression of Arg-1 mRNA and effectively inhibited relative expression of iNOS mRNA (Figure 5D, *p* < 0.001 and *p* < 0.01, respectively), which is consistent with the that of immunohistochemical staining in Figures 5B,C.

### Ginkgolide B Promotes the Production of Neurotrophic Factors From Astrocytes

**Figure 6:**
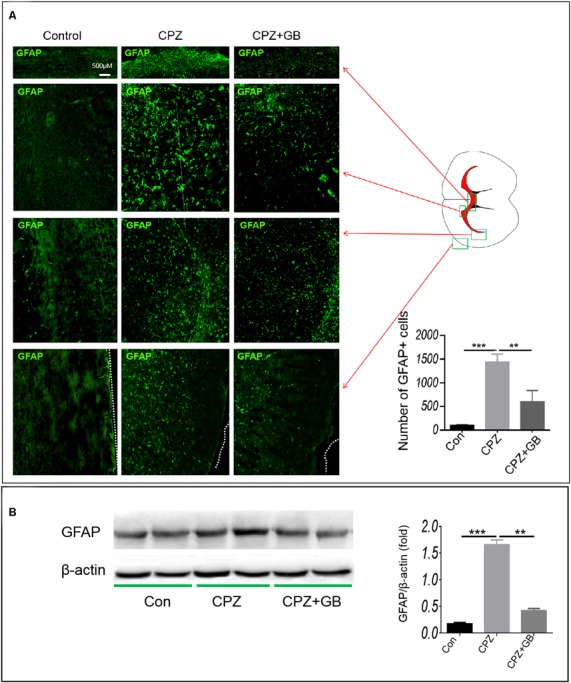
GB inhibited astrocyte response. Mice were fed with chow containing 0.2% CPZ for 6 weeks and were intraperitoneally injected with GB for 14 consecutive days from the 4th to 6th week of CPZ feeding. **(A)** Distribution of GFAP^+^ astrocytes in the corpus callosum, claustrum, cingulate cortex and cortex of the brain by immunohistochemistry, **(B)** expression of GFAP protein in the extract of the brain by western blot. Representative images are obtained from three to four mice/each group, with similar results. The results represent the mean ± SEM. **p < 0.01, ***p < 0.001.

Previous studies have shown that neurotrophic factors released by astrocytes promote myelin repair. Compared with mice fed with normal diet, CPZ feeding caused the migration and accumulation of astrocytes in the corpus callosum (Figure 6A, *p* < 0.001), which can be decreased by GB treatment (Figure 6A, *p* < 0.01). We also compared the distribution of astrocytes in other regions of the brain, finding that the numbers of GFAP^+^ astrocytes in the claustrum, cingulate cortex and cortex of mice fed with CPZ were also elevated as compared with mice fed with normal diet (Figure 6A). The results from Western blot also confirmed that CPZ feeding induced the expression of GFAP in the brain (Figure 6B, *p* < 0.001) which can also be inhibited by GB treatment (Figure 6B, *p* < 0.01). These results suggest that CPZ feeding induces the migration and activation of astrocytes, while GB treatment effectively inhibits the activation of astrocytes.

**Figure 7:**
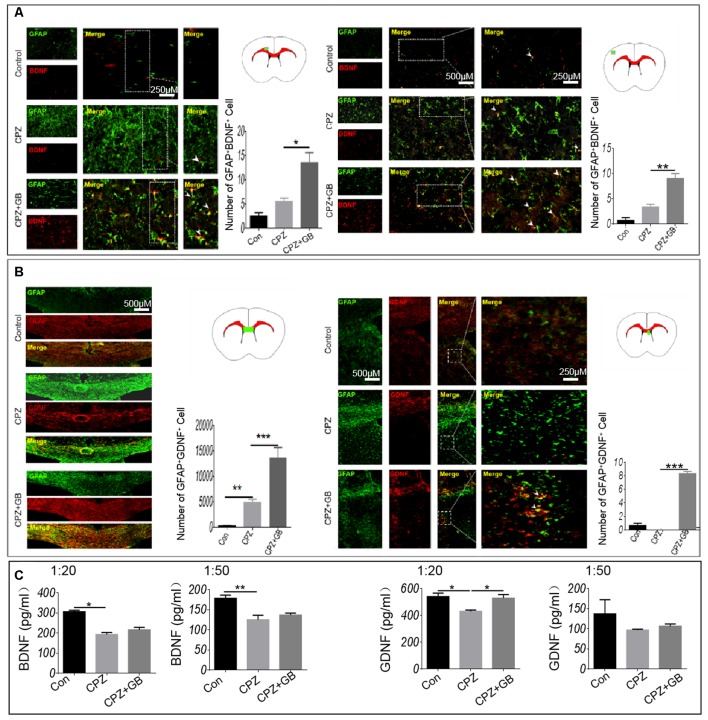
GB induced the production of BDNF and GDNF from astrocytes. Mice were fed with chow containing 0.2% CPZ for 6 weeks and were intraperitoneally injected with GB for 14 consecutive days from the 4th to 6th week of CPZ feeding. **(A)** Double immunohistochemical staining with anti-GFAP and anti-BDNF in the cingulate cortex and cortex, **(B)** double immunohistochemical staining with anti-GFAP and anti-GDNF in the corpus callosum and subventricular region. **(C)** Levels of BDNF and GDNF in the extract of the brain by ELISA. Representative images are obtained from three to four mice/each group, with similar results. The results represent the mean ± SEM. *p < 0.05, **p < 0.01, ***p < 0.001.

To further observe whether GB treatment can induce astrocytes to produce nutrient factors, contributing to the regeneration of myelin sheath, immunohistochemistry was used to detect the expression of BDNF and GDNF on GFAP^+^ astrocytes. The results showed that GB treatment effectively induced the expression of BDNF in GFAP^+^ astrocytes in the cingulate cortex and cortex, as compared with mice fed with CPZ (Figure 7A, *p* < 0.05, respectively). Similarly, although CPZ feeding can slightly induce the expression of GDNF in astrocytes (Figure 7B, *p* < 0.01), GB treatment further stimulated astrocytes to up-regulate the expression of GDNF in the corpus callosum and subventricular region (Figure 7B, *p* < 0.001). The results from ELISA found that CPZ feeding declined the levels of BDNF (1:20 and 1:50) and GDNF (1:20) in the extract of the brain (Figure 7C, *p* < 0.05 and *p* < 0.01, respectively). GB intervention slightly increased the production of BDNF and GDNF, although the difference was not statistically significant (Figure 7C).

**Figure 8:**
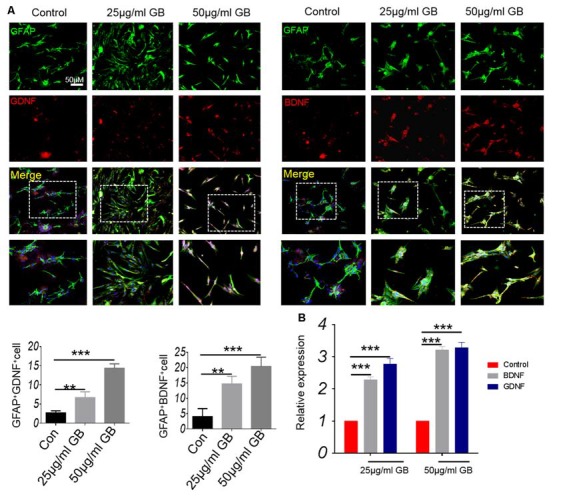
GB promoted the production of neurotrophic BDNF and GDNF in primary astrocytes. **(A)** Double immunocytochemistry staining with anti-GFAP and anti-BDNF/anti-GDNF in the primary astrocytes. **(B)** Relative expression of BDNF and GDNF mRNA in the primary astrocytes by qPCR. Representative images are obtained from three replicate samples/each group, with similar results. The results represent the mean ± SEM. **p < 0.01, ***p < 0.001.

*In vitro* cell experiments showed that GB (both 25 μg/ml and 50 μg/ml) significantly induced the expression of GDNF and BDNF that were co-localized in GFAP^+^ astrocytes (Figure 8A, *p* < 0.01, *p* < 0.001, respectively). At the same time, the results from qPCR showed that relative expression of BDNF and GDNF mRNA was elevated by GB (both 25 μg/ml and 50 μg/ml) compared with control (Figure 8B, *p* < 0.001, respectively). These results are also similar to those obtained by the *in vivo* model, indicating that GB might promote astrocytes to secrete neurotrophic BDNF and GDNF in the CPZ model.

### Ginkgolide B Promotes the Generation of Oligodendrocyte Precursor Cells

**Figure 9:**
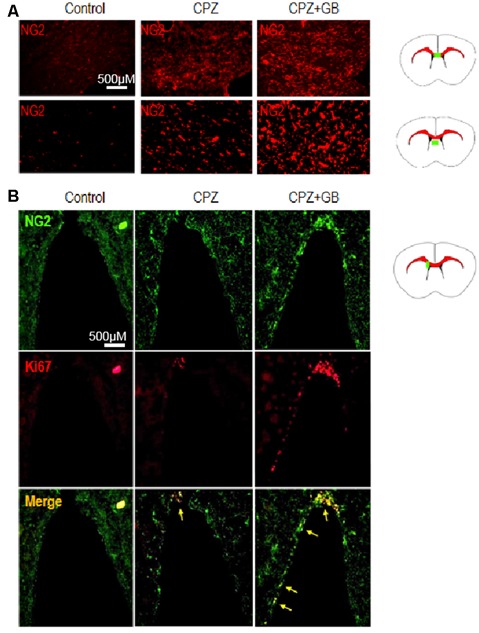
GB promoted the generation of NG2 oligodendrocyte precursor cells (OPCs). Mice were fed with chow containing 0.2% CPZ for 6 weeks and were intraperitoneally injected with GB for 14 consecutive days from the 4th to 6th week of CPZ feeding. **(A)** NG2^+^ OPCs in the corpus callosum and lateral septal nucleus of the brain by immunohistochemistry, and **(B)** double immunohistochemical staining with anti-NG2 and anti-Ki67 in the subventricular zone (SVZ; yellow arrow) by immunohistochemistry. Representative images are obtained from three to four mice/each group, with similar results.

Oligodendrocyte precursor cells (OPCs), also known as NG2 glia, remain proliferative and continuously generate myelinating oligodendrocytes. In response to a CPZ-induced demyelinating insult, NG2^+^ OPCs were rapidly generated in the corpus callosum and lateral septal nucleus of the brain (Figure 9A). However, GB treatment further promoted the generation of NG2^+^ OPCs in these regions (Figure 9A). In the subventricular zone (SVZ), NG2^+^ OPCs expressing Ki67 were obviously enhanced after GB treatment compared with CPZ-fed mice (Figure 9B, yellow arrow), revealing that GB-induced NG2^+^ OPCs are proliferating.

**Figure 10:**
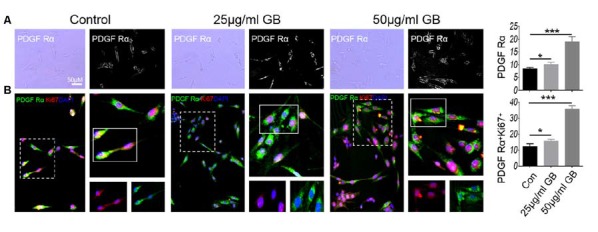
GB (both 25 μg/ml and 50 μg/ml) promoted the generation of PDGFRα OPCs. **(A)** Bright field: PDGFRα. **(B)** Double immunocytochemistry staining with anti-PDGFRα and anti-Ki67 in the PDGFRα by immunocytochemistry. Representative images are obtained from three replicate samples/each group, with similar results. The results represent the mean ± SEM. *p < 0.05, ***p < 0.001.

*In vitro* cell experiments showed that GB treatment (25 μg and 50 μg/ml) induced the formation of PDGF Ra+ OPCs (Figures 10A,B, *p* < 0.05 and *p* < 0.001, respectively). At the same time, low concentration of GB (25 μg/ml) slightly induced the expression of Ki67 by PDGFRα^+^ OPCs (Figures 10A,B, *p* < 0.05), while high concentration of GB (50 μg/ml) obviously enhanced PDGFRα^+^ OPCs expressing Ki67 (Figures 10A,B, *p* < 0.001), suggesting that GB induced the formation and proliferation of PDGFRα^+^ OPCs.

## Discussion

GB is a terpene lactone found in Ginkgo biloba extract and widely used in the treatment of various neurological diseases, including cerebral ischemia and neurodegeneration. Previous evidence has demonstrated that GB has neuroprotective and anti-inflammatory effects. Here, we observed the therapeutic effect of GB in the demyelination model induced by CPZ, manifested by the improvements in behavior and demyelination.

The occurrence and development of MS involve many pathogenic factors, including activation of T and B cells, immune triggered inflammation, mitochondrial dysfunction, cell apoptosis and so on. However, glial activation is considered to play a decisive role in the progression of MS (Lassmann, 2014; Mahad et al., 2015). Microglia modulate astrocyte phenotype and function (Rothhammer et al., 2018). The activated microglia release different cytokines (IL-1β, IL-6, and TNF-α) as well as oxidation products (NO and ROS), leading to exacerbation of inflammatory cascade and subsequent progression of disease progression (Lassmann et al., 2012; Mahad et al., 2015; Zrzavy et al., 2017). Like the microglia, the A1 subtype of astrocytes is a potent killer of neurons and oligodendrocytes in EAE models and was reported in acute and chronic MS lesions (Liddelow et al., 2017). Nevertheless, the astrocytes in progressive MS play a more specific role in the maintenance of the local inflammation. In fact, the activated microglia can induce neurotoxic reactive astrocytes that contribute to the death of neurons and oligodendrocytes in neurodegenerative disorders, and meanwhile, provide opportunities for the development of new treatments for these diseases (Liddelow et al., 2017).

In a non-immune induced demyelinating model mediated by CPZ, we observed the migration and accumulation of microglia and astrocytes toward the corpus callosum and corresponding myelin sheath regions. A demyelinating event often results not only in the loss of intact myelin sheaths but also the accumulation of myelin debris at the lesion site (Lampron et al., 2015). Inflammatory agents such as lipopolysaccharide (LPS) can stimulate both the migration and phagocytic activity of macrophages (Vallières et al., 2006). It was reported that microglia were activated with increased inflammatory cytokines and myelin phagocytosis (Shobin et al., 2017), indicating that myelin debris may be an inflammatory stimulus for inflammatory microglial activation. During initial demyelination pathology, microglia activation is beneficial since they are involved in myelin debris clearance. However, with the progression of the demyelinating lesion, activated microglia elicit detrimental effects by the overexpression of inflammatory cytokines such as IL-1β, IL-6, and TNF-α in the surrounding tissue lesion. In this study, we observed that CPZ feeding induced the inflammatory phenotype of microglia in the Corpus callosum, which should be related to the migration of microglia and phagocytosis of myelin debris. However, it is still unclear whether the decrease of microglia enrichment in the corpus callosum after GB treatment is due to the inhibition of microglia migration and activation by GB or to the acceleration of myelin debris clearance, leading to the remove of microglia from the corpus callosum and the transformation of M2 phenotype.

Previous evidence has demonstrated that astrocytes play dual and controversial roles in demyelinating diseases. The ablation of astrocytes in transgenic mice receiving CPZ causes a significant decrease in the demyelination within the corpus callosum associated with a reduction in the number of activated microglia in demyelinated sites (Skripuletz et al., 2013). The ablation of astrocytes also reduced the recruitment of microglia as phagocytosing cells to clear the damaged myelin sheath (Skripuletz et al., 2013). These results suggest that astrocytes are harmful to myelin sheath repair. However, astrocytes have the capacity to modify the constituents of the extracellular matrix in MS by producing a variety of nutritional factors that directly influence remyelination within the CNS (Clemente et al., 2013). Consistent with the above results, we found that the treatment of GB promoted the repair of the myelin sheath, accompanied by the up-regulation of BDNF and GDNF expression in astrocytes, suggesting that GB induces astrocytes to produce neurotrophic factors, which could promote the formation and differentiation of oligodendrocytes in demyelinated regions. BDNF promotes oligodendrocyte differentiation and MBP expression (Dubois-Dalcq and Murray, 2000; KhorshidAhmad et al., 2016).

At present, we do not elucidate the mechanism by which GB promotes astrocyte to produce neurotrophic factors. It is reported that activated microglia induced neurotoxic reactive astrocytes that resulted in the death of neurons and oligodendrocytes by secreting IL-1α and TNF-α (Liddelow et al., 2017). IL-1β secreted from microglia is a crucial molecule to alter the functions of astrocytes upon activation of TLR4 (Yan et al., 2019). Therefore, we speculate that GB may maintain the supporting and nutritional functions of astrocytes by inhibiting microglial inflammatory response or inducing microglial M2 polarization in this study.

Oligodendrocyte recruitment and differentiation are regulated by numerous environmental factors, including cytokines, chemokines and growth factors (Plemel et al., 2017). In particular, the balance between microglia and astrocytes should create a beneficial microenvironment, which reduces myelin damage and promotes myelin regeneration. Here, we found that GB can regulate the biological effects of microglia and astrocytes, although it inhibited the enrichment of microglia and astrocytes in the demyelinated areas. GB inhibited the inflammatory phenotype and cytokine production of microglia and induced the growth/nutrient secretion of astrocytes. The former can reduce the further oligodendrocyte damage mediated with the inflammatory microenvironment, while the latter can help the formation and differentiation of oligodendrocytes, achieving a dynamic balance of beneficial microenvironment for myelin repair. Our results also clearly demonstrate that GB treatment promoted the generation of NG2^+^ OPCs, which expressed Ki67, suggesting that GB-induced NG2^+^ OPCs are proliferative and contribute to the remyelination in CPZ-induced demyelination.

**Figure 11:**
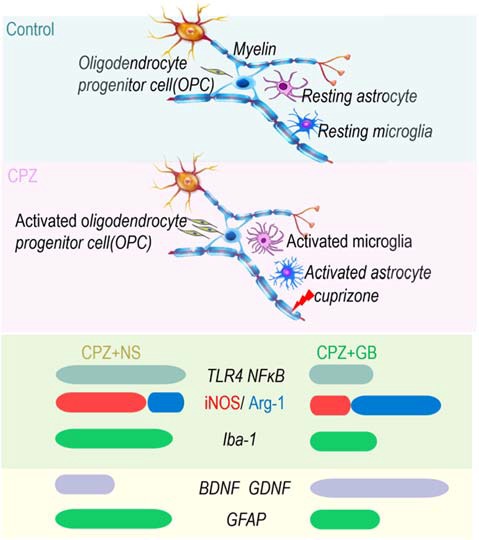
GB enhanced spontaneous remyelination by regulating the dynamic balance of microglia and astrocytes. On the one hand, GB inhibits inflammatory signals (TLR4 and NF-κB) and induces the M2 phenotype of microglia (iNOS ↓ and Arg-1 ↑); on the other hand, GB promotes astrocytes to produce BDNF and GDNF.

In conclusion, GB treatment protected from CPZ-induced behavior abnormalities, inhibited the inflammatory response, and promoted myelin generation in CPZ demyelinating model, implicating that GB might act as a novel strategy for the treatment of MS in clinic. The therapeutic potential of GB in CPZ-induced demyelination may be based on the following mechanisms of action: (i) maintains microglia in a deactivated state/M2 phenotype and avoids the deleterious effects of excessive neuroinflammation; and (ii) promotes myelin repair and regeneration by upregulating neurotrophic factors derived from astrocytes (Figure 11). These results highlight the importance of targeting microglia and astrocytes and regulating the dynamic balance of microglia and astrocytes during the remyelination processes. However, the communication and association between microglia and astrocytes mediated with GB still need to be further explored, developing advanced GB therapies to combat neuroinflammation and demyelination in MS and other related pathogenic lesions.

## Conclusion

GB promotes myelin generation by regulating the dynamic balance of microglia and astrocytes, but the precise molecular mechanisms are not fully understood, and further studies are required.

## Data Availability Statement

All datasets generated for this study are included in the article.

## Ethics Statement

The animal study was reviewed and approved by Laboratory and Ethics Committee of Shanxi University of Chinese Medicine.

## Author Contributions

J-JY conducted this project and wrote the manuscript. YH and JA carried out Western blot. QM, R-XS and QW participated in data analysis. J-ZY proofread the article. B-GX and C-GM designed the experiments.

## Conflict of Interest

The authors declare that the research was conducted in the absence of any commercial or financial relationships that could be construed as a potential conflict of interest.

## Abbreviations

MS: multiple sclerosis
CNS: central nervous system
OPCs: oligodendrocyte precursor cells
GB: Ginkgolide B
RT: room temperature.
